# Supplementary material for: Local environment in biomolecular condensates modulates enzymatic activity across length scales
Source: Nat Commun. 2024 Apr 18;15:3322. doi: 10.1038/s41467-024-47435-w (PMC11026464; doi:10.1038/s41467-024-47435-w)
Supplement: Supplementary file 1 — Supplementary Information [file 41467_2024_47435_MOESM1_ESM.pdf]

Supplementary Information for:

Local environment in biomolecular condensates modulates enzymatic activity across length scales

Marcos Gil-Garcia[1], Ana I. Benítez-Mateos[2], Marcell Papp[1], Florence Stoffel[1], Chiara Morelli[1], Karl Normak[1], Katarzyna Makasewicz[1], Lenka Faltova[1], Francesca Paradisi[2], Paolo Arosio[1]*

[1] Department of Chemistry and Applied Biosciences, Institute for Chemical and Bioengineering, ETH Zurich, Zurich, Switzerland.

[2] Department of Chemistry, Biochemistry and Pharmaceutical Sciences, University of Bern, Bern, Switzerland.

*Corresponding author

Laf1-NOX

MGSSHHHHHHSSGLVPRGSHMESNQSNNGGSGNAALNRGGRYVPPHLRGGDGGAAAAASAGGDDRRGGAGGGGYRRGGGNSGGGG
GGGYDRGYNDNRDDRDNRGGSGGYGRDRNYEDRGYNGGGGGGGNRGYNNNRGGGGGGYNRQDRGDGGSSNFSRGGYNNRDEGSD
NRGSGRSYNNDRRDNGGDGMEATLPVLDAKTAALKRRSIRRYRKDPVPEGLLREILEAALRAPSAWNLQPWRIVVVRDPATKRALREAAFG
QAHVEEAPVVLVLYADLEDALAHLDEVIHPGVQGERREAQKQAIQRAFAAMGQEARKAWASGQSYILLGYLLLLLEAYGLGSVPMLGFDPER
VKAILGLPSHAAIPALVALGYPAEEGYPSYRLPLERVVLWR

Net charge: +7

Dbp1-NOX

MGSSHHHHHHSSGLVPRGSHMADLPQKVSNLSINNKENGGGGGKSSYVPPHLRSRGKPSFERRSPKQKDKVTGGDFFRRAGRQTGNNG
GFFGFSKERNGGTSANYNRRGSSNYKSSGNRWVNGKHIPGPKNAKLQKAELFGVHDDPDYHSSGIKFDNYDNIPVDASGKDVPEPILMEAT
LPVLDAKTAALKRRSIRRYRKDPVPEGLLREILEAALRAPSAWNLQPWRIVVVRDPATKRALREAAFGQAHVEEAPVVLVLYADLEDALAHLDE
VIHPGVQGERREAQKQAIQRAFAAMGQEARKAWASGQSYILLGYLLLLLEAYGLGSVPMLGFDPERVKAILGLPSHAAIPALVALGYPAEEGY
PSYRLPLERVVLWR

Net charge: +14

Ddx4-NOX

MGSSHHHHHHSSGLVPRGSHMGDEDWEAEINPHMSSYVPIFEKDRYSGENGDNFNRTPASSSEMDDGPSRRDHFMKSGFASGRNFGNR
DAGECNKRDNTSTMGGFGVGKSFGNRGFSNSRFEDGDSSGFWRESSNDCEDNPTRNRGFSKRGGYRDGNNSEASGPYRRGGRGSFRG
CRGGFGLGSPNNDLDPDECMQRTGGLFGSRRPVLSGTGNGDTSQSRSGSGSERGGYKGLNEEVITGSGKNSWKSEAEGGESMEATLPVL
DAKTAALKRRSIRRYRKDPVPEGLLREILEAALRAPSAWNLQPWRIVVVRDPATKRALREAAFGQAHVEEAPVVLVLYADLEDALAHLDEVIHP
GVQGERREAQKQAIQRAFAAMGQEARKAWASGQSYILLGYLLLLLEAYGLGSVPMLGFDPERVKAILGLPSHAAIPALVALGYPAEEGYPSYR
LPLERVVLWR

Net charge: -1

Supplementary Figure 1. Amino acid sequence and net charge of Laf1-NOX, Dbp1-NOX and Ddx4-NOX. Grey and green backgrounds indicate the low complexity domains (LCDs) and the globular domain, respectively. The net charge at physiological pH for each construct is indicated next to the corresponding amino acid sequence.

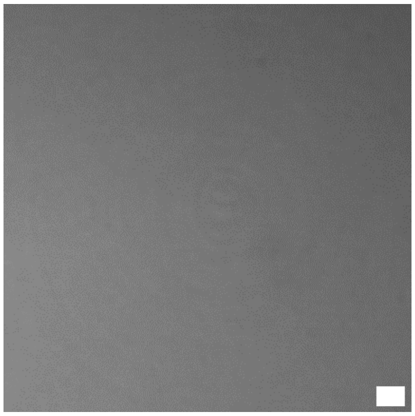

Supplementary Figure 2. Absence of micron-sized condensates in solutions of NOX. Representative confocal microscopy image of 5 µM NOX solutions. Scale bar represents 5 µm. The experiment was repeated 3 times independently with similar results.

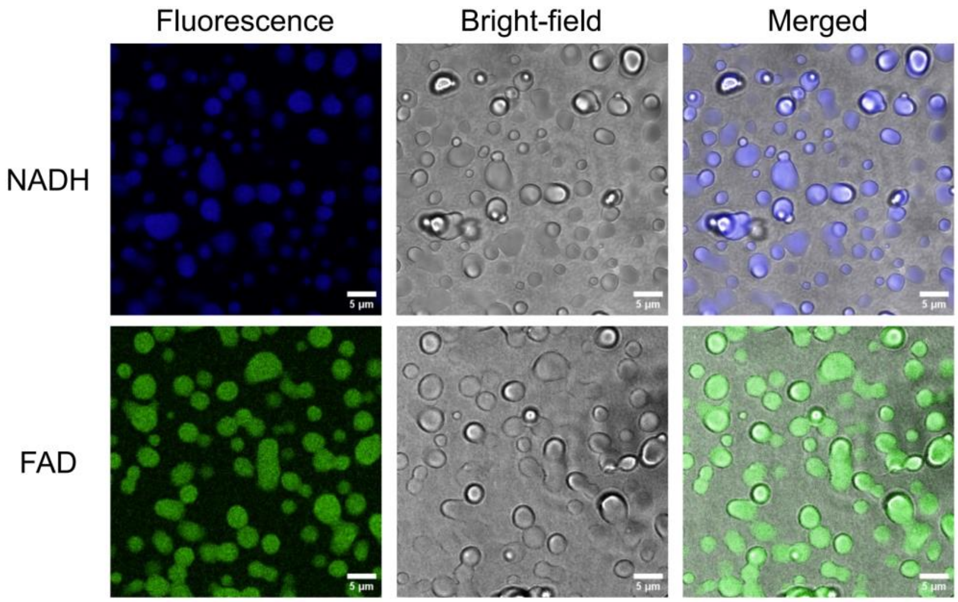

Supplementary Figure 3. Substrate and cofactor partition into Dbp1-NOX condensates. Representative fluorescence and bright-field confocal microscopy images showing the recruitment of NADH (top, blue fluorescence) and FAD (bottom, green fluorescence) in Dbp1-NOX condensates. NADH and FAD were added individually to avoid interference of the reaction. The merged images confirm the localization of substrate and cofactor within the condensates. The experiment was repeated 3 times independently with similar results.

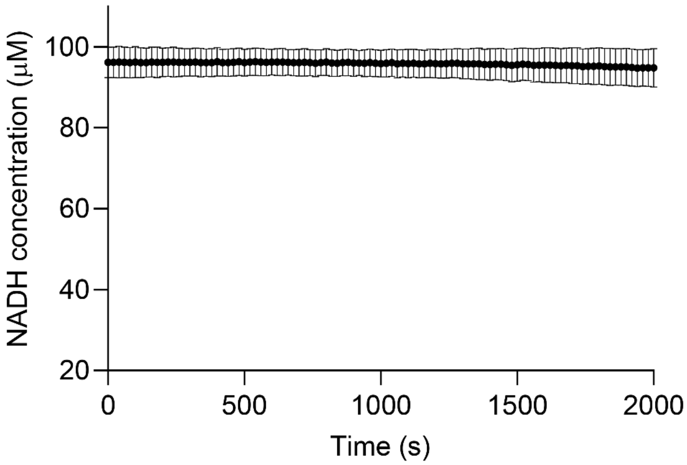

Supplementary Figure 4. Measurement of NADH concentration in the presence of Dbp1-NOX condensates in the absence of cofactor (no reaction occurring). Representative profile of the NADH concentration measured by absorbance at 340 nm during 30 min in the presence of 1 μM Dbp1-NOX condensates without cofactor. n = 3 independent experiments. Data are presented as mean values +/- SEM.

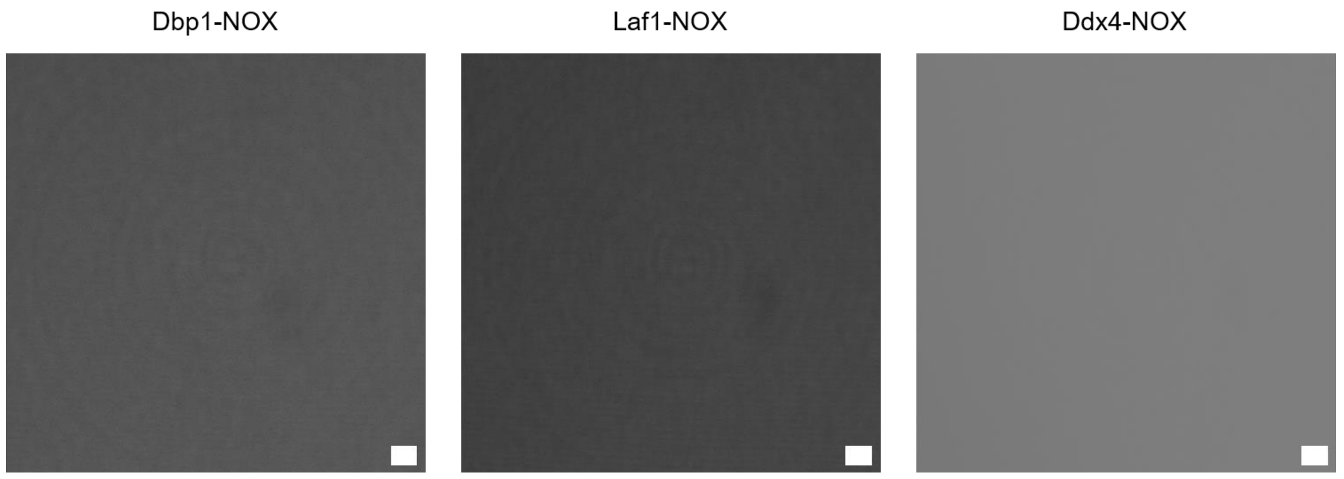

Supplementary Figure 5. Absence of micron-sized condensates at 500 mM NaCl. Representative confocal microscopy images of the homogeneous systems composed of Dbp1-NOX, Laf1-NOX and Ddx4-NOX. Scale bar represents 5 µm. The experiment was repeated 3 times independently with similar results.

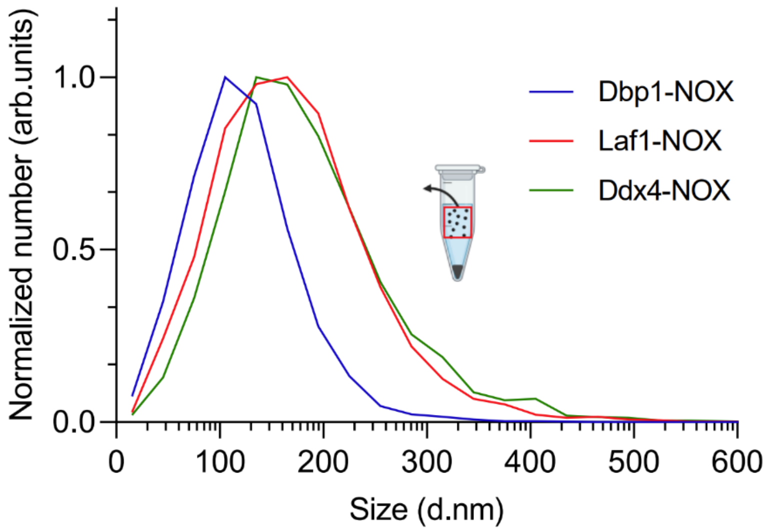

Supplementary Figure 6. Size distribution of the dilute phase after removal of the micron-sized condensates by centrifugation measured by nanoparticle tracking analysis (NTA). The results confirm the presence of nanoclusters. Created with BioRender.com.

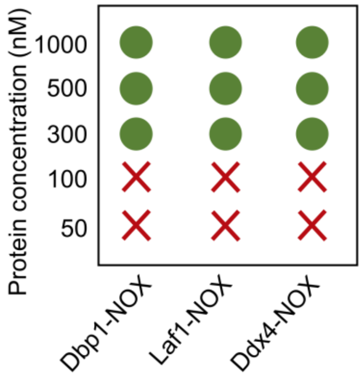

Supplementary Figure 7. Phase diagram of the different LCD-NOX fusion proteins at different protein concentration and constant ionic strength of 20 mM NaCl. Green circles and red cross indicate presence and absence of phase separation, respectively.

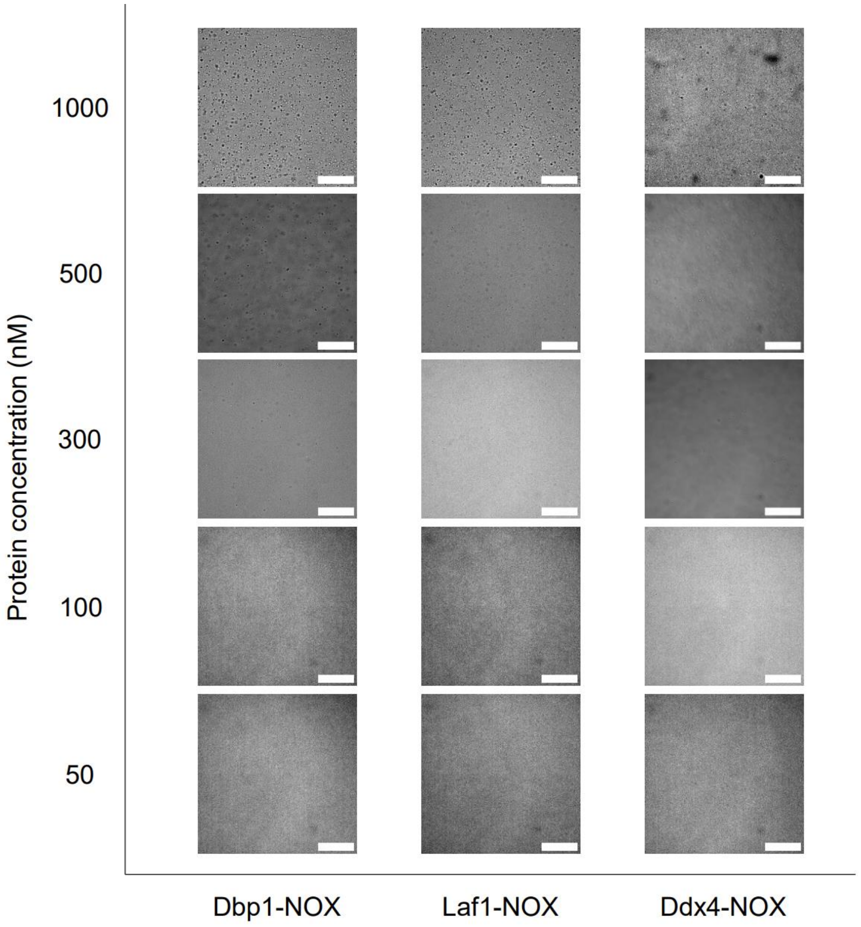

Supplementary Figure 8. Representative bright-field microscopy images of LCD-NOX samples at different protein concentration and constant ionic strength of 20 mM NaCl. Scale bar represents 50 µm. The experiment was repeated 3 times independently with similar results.

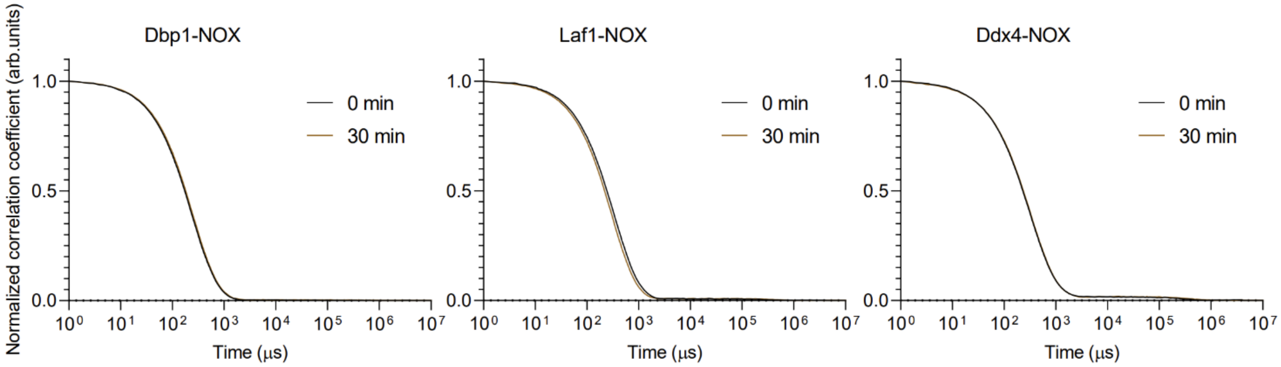

Supplementary Figure 9. Analysis of clusters size at different time points. Temporal evolution of DLS autocorrelation functions of solutions of 280 nM Dbp1-NOX, Laf1-NOX and Ddx4-NOX with 20 mM NaCl at 0 min and 30 min at room temperature.

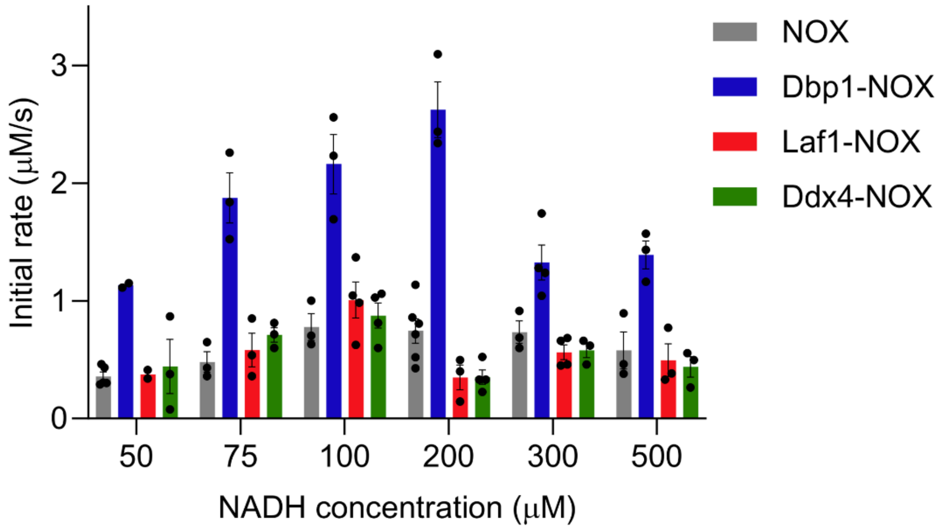

Supplementary Figure 10. Condensates composed of different chimeric proteins alter NOX enzymatic activity. Initial rates of the heterogeneous systems (in presence of condensates) and the NOX homogeneous system at different substrate concentrations. n ≥ 2 independent experiments. Data are presented as mean values +/- SEM.

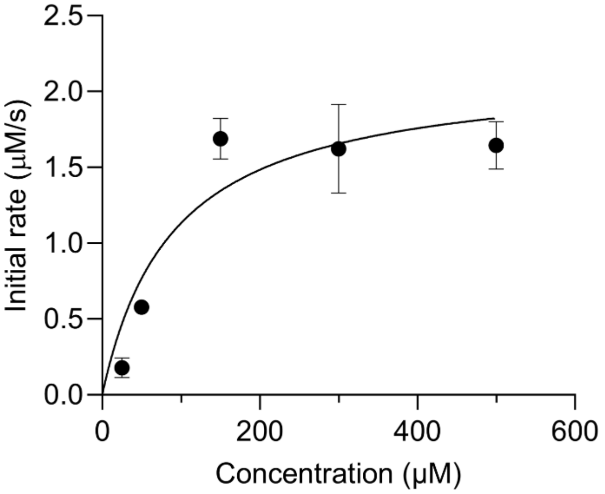

Supplementary Figure 11. Dependence of Dbp1-NOX initial rates on cofactor concentration. Initial rates of the Dbp1-NOX heterogeneous system measured by NADH absorbance at 340 nm at various FAD concentrations. n ≥ 2 independent experiments. Data are presented as mean values +/- SEM.

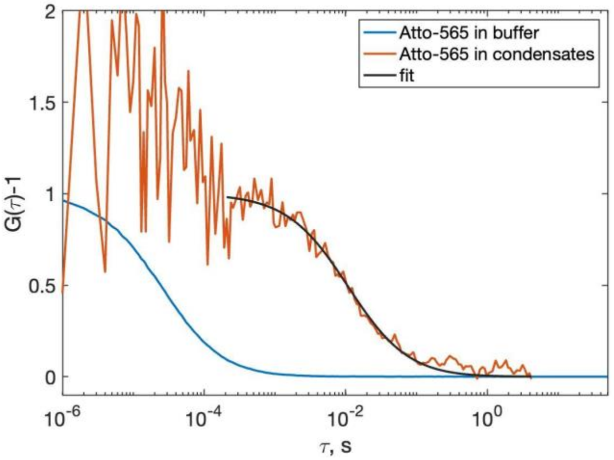

Supplementary Figure 12. Fluorescence autocorrelation curves for Atto-565 dye in buffer and within Dbp1-NOX condensates. The autocorrelation curve was fitted to a model assuming a single diffusing component and the extracted diffusion coefficient is 3.8 ± 0.5 $\mu m^2/s$.

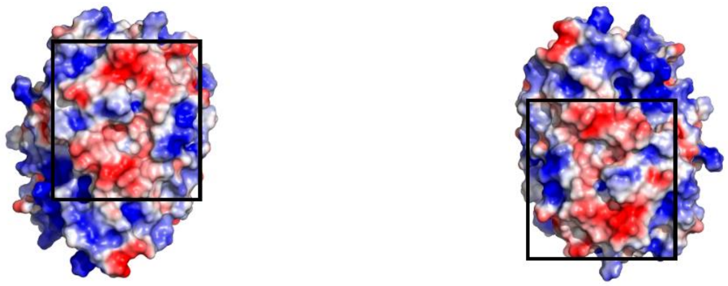

Supplementary Figure 13. Surface electrostatic representations of dimeric NOX (PDB 1NOX). Residues were colored according to their anionic (red) or cationic (blue) character using the APBS electrostatics plugin in PyMOL. The anionic patches are indicated by a black square.

Supplementary Table 1. Numerical values used for the kinetic modelling.

Protein concentration	Φ_1	Φ_2	Φ_3	r* (µM/s)	r_1 (µM/s)	r_2 (µM/s)	r_3 (µM/s)	E_1 (µM)	E_2 (µM)	E_3 (µM)	r_1/E_1** (1/s)	r_2/E_2* (1/s)	r_3/E_3* (1/s)
20 nM	1	-	-	-	0.011<br>0.015<br>0.017<br>0.022	-	-	0.02	-	-	0.57<br>0.73<br>0.85<br>1.12	-	-
280 nM	1	$2.61*10^{-5}$	-	0.44<br>0.56<br>0.88<br>0.93	0.10<br>0.12<br>0.14<br>0.19	$1.32*10^4$<br>$1.66*10^4$<br>$2.81*10^4$<br>$2.88*10^4$	-	0.168	4290	-	0.57<br>0.73<br>0.85<br>1.12	3.07<br>3.87<br>6.54<br>6.71	-
1 µM	1	$2.61*10^{-5}$	$1.52*10^{-4}$	1.13<br>1.87<br>2.16<br>2.62	0.10<br>0.13<br>0.15<br>0.20	$1.41*10^4$<br>$1.78*10^4$<br>$3.01*10^4$<br>$3.09*10^4$	$4.35*10^3$<br>$8.41*10^3$<br>$8.06*10^3$<br>$10.59*10^3$	0.180	4600	4600	0.57<br>0.73<br>0.85<br>1.12	3.07<br>3.87<br>6.54<br>6.71	0.95<br>1.83<br>1.75<br>2.30

*r = average rate with contribution of both dilute and dense phase. The four values correspond to the four concentrations of substrate: 50, 75, 100 and 200 µM

**the r_1/E_1 values were calculated by assuming the same K_M and k_{cat} of the NOX enzyme at 280 nM
